# A rare case of reduction en masse of incarcerated inguinal hernia: A case report

**DOI:** 10.1016/j.ijscr.2024.110112

**Published:** 2024-08-06

**Authors:** Mohsen Najmaddini, Arvin Mirshahi, Mohammad Mehdi Shadravan, Nafise Zangane, Mahyar Mohammadifard, Siavash Kafian Atary

**Affiliations:** a Department of Surgery, School of Medicine, Birjand University of Medical Sciences, Birjand, Iran; b Students' Scientific Research Center, Tehran University of Medical Sciences, Tehran, Iran; c Student Research Committee, Shahid Beheshti University of Medical Sciences, Tehran, Iran; d Student Research Committee, Birjand University of Medical Sciences, Birjand, Iran; e Department of Radiology, School of Medicine, Birjand University of Medical Sciences, Birjand, Iran

**Keywords:** Inguinal hernia, Incarcerated inguinal hernia, Reduction en masse, Surgery, Iran, Case report

## Abstract

**Introduction and importance:**

Reduction en masse is a rare complication of incarcerated inguinal hernias, occurring when the herniated sac, along with the trapped hernia, returns to the preperitoneal space.

**Case presentation:**

In this study, we describe a 74-year-old male patient who presented to the hospital with a history of manual hernia reduction and complaints of nausea, vomiting, and constipation for two weeks. After undergoing paraclinical tests, he underwent open surgery with a diagnosis of hernia reduction en masse, during which the hernia sac was separated from the surrounding structures. Abdominal and peritoneal defects were also repaired intra-abdominally. After his condition stabilized, the patient was discharged with prescription medications.

**Discussion:**

Reduction en masse in inguinal hernia cases is rare, where the hernia sac and intestinal contents are reduced while the intestine remains incarcerated. Computed tomography (CT) scans can aid in diagnosis, revealing characteristic features such as closed-loop obstruction and inguinal soft tissue changes. Treatment options include open laparotomy and laparoscopy, with laparoscopy being preferred depending on surgeon expertise, assessment of intestinal viability post-reduction, and patient stability.

**Conclusions:**

The potential complications of hernia reduction en masse should be emphasized to patients who choose not to remain under medical supervision, as well as to physicians and surgeons when patients re-present following manual hernia reduction. Increasing awareness about this condition at relevant times is crucial.

## Highlights

• Incarcerated inguinal hernias can lead to a rare complication called reduction en masse.
• Red flags for reduction en masse include abdominal pain, nausea, vomiting, and absence of fecal or gas excretion.
• CT findings for reduction en masse include closed-loop obstruction, circular funicular structure at obstruction site, presence of bladder beak, and unilateral inguinal soft tissue appearance.
• The potential complication of hernia reduction en masse should be highlighted to patients and physicians, especially after manual reduction.

## 1 Introduction and importance

Reduction en masse is a rare complication of incarcerated inguinal hernias [1]. Incarceration is a condition where external hernias do not reduce back into the abdomen, thereby increasing the risk of intestinal obstruction, strain, and strangulation [2]. Delayed detection of incarceration may result in necrosis of the incarcerated intestine [3]. The incidence of this complication is approximately 1 in 13,000 hernias [4]. As a sporadic event, reduction en masse can be difficult to diagnose before surgery, and its definitive surgical treatment is unclear [5]. However, a computed tomography (CT) scan and a thorough medical history are useful for an accurate preoperative diagnosis of this complication [6]. This study describes a 74-year-old male patient who presented to the hospital with complaints of nausea, vomiting, and constipation for two weeks. After undergoing paraclinical tests, he underwent open surgery with a diagnosis of hernia reduction en masse. This case report follows the Surgical CAse REport (SCARE) and Consensus Preferred Reporting Of CasE Series in Surgery (PROCESS) guidelines [7,8].

## 2 Case presentation

**Fig. 1:**
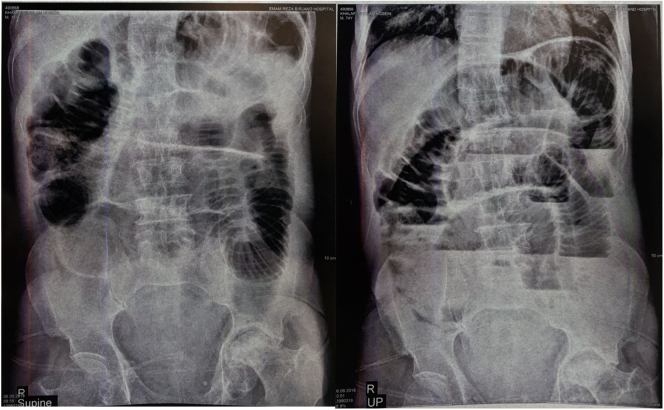
Supine (AP) and upright (PA) radiographs of the abdomen showing visible dilatation and multiple fluid levels in the intestine, suggesting obstruction.

**Fig. 2:**
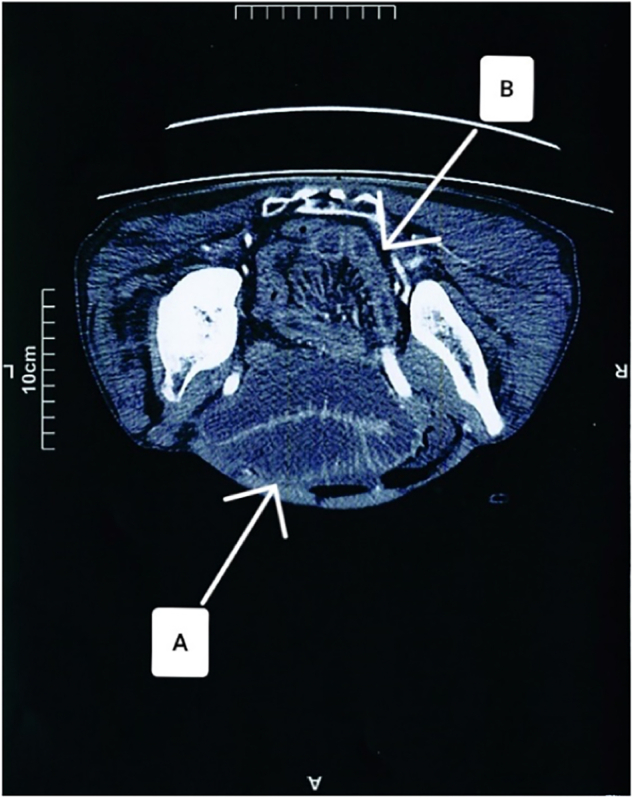
CT scan of the patient's abdomen; Flash A indicates dilated bowel loops, Flash B indicates normal bowel loops.

A 74-year-old man presented with a history of hypertension (HTN), chronic obstructive pulmonary disease (COPD), and oral opium addiction. He had also undergone left-sided inguinal hernia repair three years previously. One month prior to the current visit, he developed a right-sided inguinal hernia, which could be reduced manually. However, on the morning of the visit, the bulge did not reduce manually, whereby the patient was referred to the hospital. An intravenous (IV) line and fluid therapy were immediately administered, and the hernia was reduced using the taxis maneuver. However, after the hernia was reduced, despite the medical team's advice to undergo surgery, the patient was discharged with personal consent and did not continue treatment. After discharge, the patient experienced abdominal pain, constipation, and vomiting twice, although there was flatus excretion. Consequently, the patient returned to the hospital one week later for continued treatment and a definitive diagnosis. On initial examination, the patient's abdomen was soft, with no (rebound) tenderness or hernia; no bloody stool or melena was observed on the rectal examination. The patient had no perianal pain but complained of anorexia, nausea, and vomiting. Since the patient had no visible abdominal mass and showed signs of small bowel obstruction on the abdominal X-ray (Fig. 1), a CT scan was requested. The CT scan revealed dilated intestinal loops (Fig. 2); thus, the diagnosis was hernia reduction en masse, given the patient's history of hernia with mass reduction one week prior to referral.

**Fig. 3:**
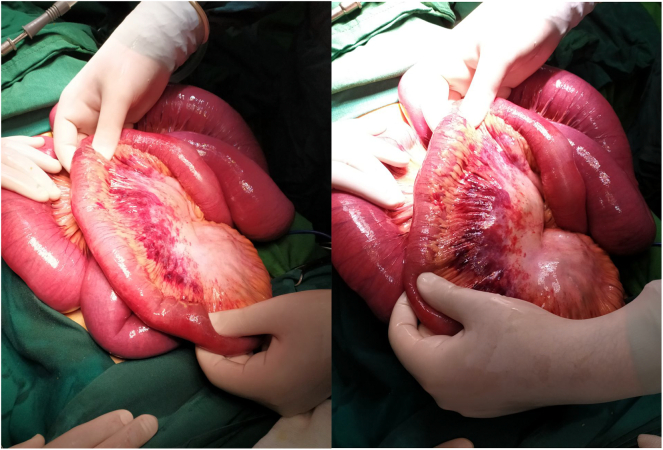
Part of the intestine trapped in the sac and discolored.

Given the patient's age and respiratory issues, the aneasthesia team recommended that laparoscopic surgery not be performed. They believed that the gas entering the abdomen could exacerbate the patient's condition and respiratory problems. The patient underwent spinal anesthesia, and a lower midline incision was made. The small bowel was dilated in the proximal portion and was found within the right inguinal hernia sac. There was a sac in the abdomen with no swelling in the inguinal area. The contents of the hernia sac were discolored, so the sac was incised, and the contents were removed (Fig. 3). The hernia sac was separated from the surrounding structures, and abdominal and peritoneal defects were repaired intra-abdominally. The suture site was repaired using a proline suture. No mesh was used to repair the defect, and the repair was done through the abdomen. Hemostasis was checked, and the abdominal layers were closed. Subsequently, the patient was transferred to the intensive care unit (ICU). The surgical procedure was performed by an attending surgeon and assistant professor at Birjand University of Medical Sciences. One day after surgery, the patient's constipation was restored, and no further problems were observed. On the second day, the patient initiated an oral ingestion regimen and tolerated it well. The patient was transferred from the ICU to the surgical ward after his condition stabilized. On the fourth day post-surgery, the patient was discharged with prescriptions for antibiotics, laxatives, and analgesics.

After the patient was discharged, long-term follow-up showed no signs of hernia recurrence or related complications. The patient was monitored for 12 months and reported no abdominal pain, nausea, or other gastrointestinal symptoms. Regular check-ups confirmed the success and durability of the surgical intervention.

## 3 Discussion and conclusion

The occurrence of reduction en masse is infrequent in cases of inguinal hernia [1]. This complication arises when the hernia sac, along with the contents of the intestine, is reduced into the abdomen while the intestine remains incarcerated in the sac [9]. This can be caused by the manual reduction of the hernia [10], as in the case of our patient, where manual reduction entrapped intestinal loops within the sac. Repetitive inguinal herniation and its reduction can cause fibrotic changes at the hernia orifice, leading to a narrow neck that makes it difficult for the bowel to withdraw from the sac [11]. The cause of this particular condition is still not well understood, but logically, fibrotic changes can develop in both bowel and sac tissue due to prolonged asymptomatic incarceration. As a result, the bowel becomes trapped and cannot move freely within the sac. Although the neck is not tight enough to cause ischemia, fibrotic changes can still occur in the neck region. Eventually, the neck becomes tighter, and ischemia begins as fibrotic changes persist [12].

Mings et al. conducted a review of more than 200 cases of en masse hernia in 1965. Although a few cases have been reported since then, the frequency of such cases remains low [13]. To the best of our knowledge, only two similar cases [12,14] have been reported in Iran, which may indicate that the physician's initial diagnosis of the patients was incorrect. Therefore, reduction en masse should be considered as a differential diagnosis if a patient presents with these symptoms after hernia reduction. However, sometimes the symptoms may be delayed, appearing up to three years later, as reported in Parvey et al.'s study [9].

After reviewing the literature in PubMed and Google Scholar databases, we conducted a comprehensive diagnostic and therapeutic review of 9 cases of reduction en masse as a rare condition. The findings from these 9 case reports were compared with our patient's case and are summarized in Table 1 [[4], [5], [6],^11^,^12^,[14], [15], [16], [17]].

**Table 1:** Comparison of clinical, diagnostic, and surgical aspects in cases of inguinal hernia reduction en masse.

First author	Year	Sex/age	History of repetitive inguinal hernia	Symptoms	Ph/E	CT-scan findings	Surgical technique	Surgical procedure and findings	Recovery and long-term follow-up	R
Najmaddini	Our case	M-74 yo	Right-side inguinal hernia for 1 month	Abdominal pain, constipation, and vomiting	No abdominal mass or tenderness	Dilated intestinal loops	Open surgery	Reduction of hernia sac, no ischemia	Monitored for 12 months without recurrence or complications	–
Oshidari	2022	M-48 yo	Left inguinal hernia	Vague periumbilical pain, markedly reduced defecation	No abdominal tenderness	High-grade small bowel obstruction due to a closed loop around the left internal inguinal canal	Laparoscopic relief using TAPP technique	Ball-like mass of jejunal loop near left internal inguinal canal, slightly bluish bowel loop but viable	Discharged two days after surgery without complications	[12]
Yano	2022	M-10 m	Bilateral open inguinal hernia repair at 4 months old	Bulge in the groin, persistent vomiting post-reduction	Abdominal distention	Protrusion of the small bowel with a closed-loop in the left groin	Laparoscopic reduction and repair	Incarcerated small bowel showed no signs of ischemia, high ligation of the hernia sac	Uneventful postoperative course	[15]
Alqassab	2022	F-62 yo	No prior hernia history mentioned, presented with acute incarceration over past 24 h	Increasing pain over the past 24 h, associated nausea and vomiting, passage of flatulence but not stool	Six-cm incarcerated left inguinal hernia, irreducible	Mechanical small bowel obstruction, incarceration of small bowel within the reduced sac	Initial open mesh repair, followed by emergent laparoscopic management	Bowel loop incarcerated in reduced hernia sac, reduced by incising peritoneum, hernia sac invaginated and closed	Discharged day 3 postoperatively after laparoscopic management	[16]
Najjari	2021	M-50 yo	Right inguinal hernia	Periumbilical pain radiating to the right lower quadrant, inability to pass gas or stool, bilious vomiting	Periumbilical and right lower quadrant tenderness	Hernia sac and its contents inside the abdominal wall, with signs of small intestine obstruction	Lichtenstein repair with mesh	Adherence to the wall of the hernia sac, slight discoloration of the intestinal loop	Monitored for 5 days and discharged without complications	[14]
Baik	2019	M-76 yo	4-year history of repeated left inguinal hernia	Abdominal pain for 2 h before admission	Increased bowel sound and tenderness in the whole abdomen	Small-bowel obstruction with a closed-loop obstruction showing a 6.2-cm oval-shaped sac in the preperitoneal space	Surgical reduction using TAPP technique	Incarcerated bowel was viable, and fluid was found in the hernia sac	Recovered without complications, discharged on the third day	[11]
Cao	2019	M-58 yo	Right inguinal hernia for about 2 years	Abdominal pain and vomiting	Soft and flat abdomen with no tenderness, no lump over right groin	Closed loop obstruction with a ball-like bowel loop near the right inguinal fossa	TAPP hernioplasty with prosthetic mesh	Right indirect hernia with thickened orifice, slightly bruised but viable bowel loop	Resumed oral intake on postoperative day 2, discharged uneventfully 6 days after operation	[4]
Arima	2018	M-62 yo	Right inguinal hernia for several years	Severe abdominal pain and difficulty in self-reduction	Persistent abdominal pain after successful reduction by physician	Closed loop of small bowel around the right inguinal region and beaked bladder	Modified Kugel™ Patch with laparoscopic observation	Fibrosed neck of hernia sac causing strangulation, Incarcerated bowel gently dragged out, mild ischemic change, no bowel resection necessary	No recurrence observed during 12-month follow-up	[5]
Yatawatta	2017	M-55 yo	Right inguinal hernia for 15 years, self-reduced	Persistent lower abdominal pain 12 h after self-reduction	Localized peritonitis, firm globular structure arising from right inguinal region	Confirmed reduction en masse of right inguinal hernia, strangulated ileum of 10 cm	Lower midline laparotomy, resection and end-to-end anastomosis	Strangulated ileum reduced, internal inguinal ring approximated with sutures	Uneventful recovery, discharged on postoperative day 5, no recurrence during follow-up	[17]
Hoshino	2015	M-61 yo	Left inguinal hernia	Vomiting and abdominal pain	Soft abdomen with some distension and tenderness over lower abdomen	Ball-like lesion containing an incarcerated bowel loop over left pelvis	Laparoscopic TAPP hernioplasty with polyester mesh	Incarcerated bowel naturally released, small intestine congestion noted but not strangulated, polyester mesh used	Uneventful recovery, discharged 4 days after surgery, no recurrence at 23-month follow-up	[6]

Abbreviations: Ph/E = physical examination; CT = computed tomography; R = references; M = male; F = female; yo = years old; m = months; TAPP = transabdominal preperitoneal; PDS = polydioxanone suture.

Diagnosing a reduction en masse of an inguinal hernia can be challenging because it is uncommon but has specific CT findings. In the vast majority of cases, CT scans are used by medical professionals for diagnosing. Kitami et al. described the CT findings associated with reduction en masse. These include closed-loop obstruction with a ball-like bowel loop, a location near the inguinal fossa, a circular funicular structure at the site of obstruction, the presence of a bladder beak along the closed loop, and the appearance of a noticeable unilateral inguinal soft tissue [18]. In 2019, Baik et al. presented a case of en masse reduction. They noted that although the clinical symptoms of these patients are not specific, CT scan imaging can show a distinctive feature called the preperitoneal hernia sac sign. In this specific feature, the hernia sac, which contains an incarcerated bowel, is located in the preperitoneal space of the lower quadrant near the inguinal fossa [11]. Characteristic CT findings were present in our case, enabling a straightforward diagnosis of reduction en masse and facilitating prompt corrective surgery.

Furthermore, for the treatment and also diagnosis of this type of hernia, open laparotomy and laparoscopy are two alternatives, with laparoscopic surgery receiving more attention in recent reports. However, several factors are important when considering laparoscopic surgery as the procedure of choice for patients with reduction en masse hernia. These include the surgeon's experience, especially for treatment, which involves careful observation and removal of non-viable parts of the intestine and closure of the hernia sac [17], the difficulty of using a laparoscope to check the viability of the intestines after reducing an incarcerated hernia [6] and the patient's condition and stability, as the patient must be stable enough to undergo laparoscopic surgery [17].

What matters, according to our patient and other cases, is that all entrapped hernias should be repaired as soon as possible, and maintenance therapy is not recommended. Confirming the hernia sac and sifting through it is of particular importance. Repairing the hernia and resolving inguinal swelling does not necessarily indicate no obstruction, as demonstrated in our patient's case.

The potential complication of hernia reduction en masse should be emphasized to patients who choose not to remain under medical supervision, as well as to physicians and surgeons when patients re-present following manual hernia reduction. Raising awareness about this condition at relevant times is crucial.

## Abbreviations

HTN: Hypertension
CT: Computed tomography
COPD: Chronic obstructive pulmonary disease
IV: Intravenous
ICU: Intensive care unit

## Patient perspective

After the surgery, I felt much better, and my abdominal pain and nausea were gone. The doctors took great care of me, and the follow-up visits were very helpful. I am very grateful for the timely treatment and continuous support.

## Consent for publication

Written informed consent for publication of clinical details and accompanying images was obtained from the patient.

## Ethical approval

Not applicable.

## Funding

None.

## Guarantor

Siavash Kafian Atary.

## CRediT authorship contribution statement

MN managed the surgery, MM interpreted CT scans and X-rays, AM, MMS, and NZ drafted the manuscript and SK revised and approved the final manuscript. All authors contributed to refining the case report and are responsible for all aspects of the work.

## Declaration of competing interest

The authors declare that they have no competing interests.

## Data Availability

The data and materials, including all the surgical images, are included in the article.
